# Inhibition of ADAM17 attenuates high glucose-induced angiogenesis and inflammation in endothelial cells partly through down-regulation of GRO-α/CXCR2 expression: implications in peritoneal dialysis

**DOI:** 10.1007/s10157-024-02546-y

**Published:** 2024-09-21

**Authors:** Na Jiang, Hao Feng, Weizhen Xie, Leyi Gu, Wei Fang, Tingting Ding, Jiangzi Yuan

**Affiliations:** 1 Department of Nephrology, Molecular Cell Lab for Kidney Disease, Shanghai Peritoneal Dialysis Research Center, Ren Ji Hospital, Uremia Diagnosis and Treatment Center, Shanghai Jiao Tong University School of Medicine, Shanghai, China; 2 Department of Nephrology, Baoshan Site of Renji Hospital, Shanghai Jiao Tong University School of Medicine, Shanghai, China

**Keywords:** Peritoneal angiogenesis, Peritoneal dialysis, ADAM17, Inflammation

## Abstract

**Background:**

Angiogenesis and inflammation are key events leading to peritoneal morphologic alteration and ultrafiltration failure in patients undergoing peritoneal dialysis (PD). The current study aims to explore the role of ADAM17 in the angiogenetic and inflammatory responses of endothelial cells.

**Methods:**

Human umbilical vein endothelial cells (HUVECs) were cultured and treated with a high glucose-containing medium. In parallel experiments, the expression of ADAM17 in HUVECs was inhibited by SiRNA interference. The mRNA and protein expression of ADAM17, GRO-α and CXCR2 were assessed by qPCR and Western blotting, respectively. The concentrations of GRO-α, VEGF, IL-6 and TNF-α in the cellular supernatants were determined by ELISA. Tube formation and migration of HUVECs were evaluated by Matrigel and transwell migration apparatus.

**Results:**

High glucose increased the expression of ADAM17, CXCR2 and GRO-α in cultured HUVECs. RNA silencing of ADAM17 abolished high glucose-mediated increase of GRO-α and CXCR2, which were accompanied by reduced secretion of VEGF, IL-6, TNF-α, as well as tube formation and cell migration in HUVECs.

**Conclusions:**

Inhibition of ADAM17 ameliorates high glucose-induced angiogenic and inflammatory responses in endothelial cells partly through down-regulation of GRO-α/CXCR2 expression.

**Supplementary Information:**

The online version contains supplementary material available at 10.1007/s10157-024-02546-y.

## Introduction

Peritoneal dialysis (PD) utilizes the peritoneum as a semi-permeable membrane to clear solute wastes and excessive fluid from the circulation [1]. Thus, successful PD relies on the structural and functional integrity of the peritoneal membrane. However, long-term exposure to bioincompatible dialysis solution, which contains a high content of glucose and glucose degradation products, causes chronic damage to the peritoneum [2]. Inflammation, angiogenesis and fibrosis are key molecular events in progressive peritoneum structural remolding [2]. It is currently believed that angiogenesis and inflammation interact with each other, as inflammatory mediators promote angiogenesis, which in turn, contributes to inflammation by enhancing the recruitment of immune cells. Thus, angiogenesis and inflammation work together and ultimately result in functional impairment of ultrafiltration and solutes clearance of the peritoneum following PD [3, 4]. However, the underlining mechanisms have not been fully elucidated.

A disintegrin and metalloproteinase (ADAM) family belongs to the metzincin subfamily of metalloproteinases. ADAM 17, also known as tumor necrosis factor-α converting enzyme (TACE), is a zinc-dependent transmembrane multidomain metalloproteinase [5]. It intensively participates in the cleavage of a number of substrates including ligands of the epidermal growth factor receptor, adhesion molecules, proinflammatory cytokines, chemokines and their receptors. So it has been reported that ADAM17 plays a critical role based on its proteolytic activity in a variety of physiological and pathological processes, such as angiogenesis, inflammation, fibrosis, tumorigenesis, and immunity [6]. Chemokine growth-related oncogene-alpha (GRO-α) and its receptor CXC chemokine receptor 2 (CXCR2) were also shown to be participated in angiogenesis and inflammation [7–9]. There is evidence showing that GRO-α/CXCR2 expression can be regulated by ADAM17 and their interaction is essential for modulation of inflammation [10, 11].

Early studies found that ADAM17 contributed to endothelial cell proliferation, migration and in vitro angiogenesis [12]. Later on, Caolo and co-researchers reported that ADAM17 activity was required for mouse retina angiogenesis in vivo [13]. However, the function of ADAM17 and GRO-α/CXCR2 axis in the pathogenesis of PD-related peritoneal angiogenesis and inflammation has not been explored so far. In the current study, we investigate the role of ADAM17 in angiogenic changes and inflammatory reactions of endothelial cells in response to high glucose condition in vitro.

## Materials and methods

This is an in vitro study conducted in cultured human umbilical vein endothelial cells (HUVECs, EA.hy 926 cell line, Chinese National Collection of Authenticated Cell Cultures).

### Cell culture and treatments

HUVECs were cultured in M199 culture medium (Hyclone) containing 10% fecal calf serum (FCS) and 10 ng/ml of epithelial cell growth factor (Promega, Madison, WI, USA). Cell passages of 5–10 were used for experiments. HUVECs were treated with medium supplemented with 2.5% d-Glucose (Sigma, USA) or 2.5% d-Mannitol (Sigma, USA) for 24 h after growth arrest for 12 h. In parallel experiments, HUVECs were incubated with serum-free medium (SFM) as a control.

### ADAM17 gene knockdown

The gene expression of ADAM17 in HUVECs were inhibited by using small interference RNA (siRNA). In brief, HUVECs were seeded into 6-well culture plates and the cellular supernatants were discarded and changed to serum-free OPTI-MEM medium (Gibco, USA) when the cells got 70% confluent. Consequently, predesigned ADAM17 or scramble siRNA (Supplementary Table 1, GenePharma, Shanghai) were added into the cell supernatant together with an equal volume of lipofectamine 3000 transfection reagent (Thermo Fisher, USA) and incubated for 12 h before further experiments. In parallel experiments, HUVECs were incubated with an equal volume of transfection reagent as control.

### Western blotting analysis

Cell lysates from control and treated HUVECs were denatured and subjected to SDS-PAGE polyacrylamide gels for electrophoreses to determine the protein expression of ADAM17, GRO-α and CXCR2. Samples were transferred onto nitrocellulose membranes by electroblotting and then probed with primary antibodies to the targeted proteins followed by the respective secondary antibodies. Bands were visualized with ECL, semi-quantitated by densitometry using ImageJ software. The expression of ADAM17, GRO-α and CXCR2 were expressed as arbitrary densitometric unit (DU) and normalized to GAPDH. Primary antibodies to ADAM17, GRO-α and CXCR2 were purchased from Abcam. Primary antibody to GAPDH and horseradish peroxidase (HRP) conjugated secondary antibodies were purchased from Beyotime biotechnology of Shanghai.

### Quantitively PCR

The messenger RNA (mRNA) expression of ADAM17, GRO-α, CXCR2, interleukin-6 (IL-6) and tumor necrosis factor-α (TNF-α) in control and treated HUVECs were determined by quantitively PCR (qPCR). Total RNA of the cells was extracted using an RNAiso Plus kit (Takara, Dalian, China) following the manufactory’s instructions. One microgram of total RNA was reverse-transcribed to cDNA using the PrimeScript RT Master Mix kit (Takara, Dalian, China). The qPCR reactions were performed in triplicate using SYBR Premix Ex Taq II (Tli RNaseH Plus; Takara, Dalian, China) and analyzed with a Light Cycler 480 (Roche). Primers used for PCR amplification of the studied genes were listed in Supplementary Table 2 (Takara, Dalian, China). The delta-delta Ct method was used to calculated the relative changes in mRNA and normalized to the housekeeping gene of GAPDH.

#### ELISA

The levels of GRO-α, vascular endothelial growth factor (VEGF), IL-6 and TNF-α in the cell supernatants were measured using commercial ELISA kits following the manufactory’s instructions. All ELISA kits were purchased from R&D Systems (USA).

### Tube formation of HUVECs

Tube formation of HUVECs was evaluated by Matrigel (Becton Dickinson, Biosciences, Franklin Lakes, NJ, USA) as previously reported [14]. In brief, a 24-well culture plate was coated with 200 μl of Matrigel in each well. HUVECs diluted in 1 ml of complete or experimental medium (at a density of 5 × 10^4^/ml) were seeded on Matrigel and incubated at 37 °C for 24 h. The formation of tube-like structures was observed under reverted phase-contrast microscopy at × 100 magnification. Five randomly chosen fields were viewed and complete ring structures created by 3 to 5 endothelial cells were considered as one tube.

### HUVECs migration

Cell migration was assessed using a transwell migration apparatus (24 wells, 6.5 mm internal diameter, 8 μm pore size; Corning, Lowell, MA, USA) as previously reported [14]. First, 10 [5] of HUVECs were suspended in 100 μl of complete medium and seeded into the upper chamber. The lower side of filter was coated with 0.1 mg/ml of gelatin. Control or experimental medium was added to the lower chamber and the system was incubated at 37 °C in an atmosphere of 95% air and 5% CO_2_ for 24 h. Then cells were fixed and stained with hematoxylin. Cells on the top of the filters were wiped off, filters were mounted, and migrated cells attached to the bottom of the filter were counted in five randomly chosen fields at × 100 magnification.

### Statistical analysis

All experiments were repeated three times and so the data were expressed as mean ± SD of three samples. One-way analysis of variance (ANOVA) coupled with post hoc test of Bonferroni were conducted to compare the differences among groups. *P* values less than 0.05 were considered as statistically significant. All the analyses were performed using Prism 6.0 (GraphPad Software, Inc., California, USA).

## Results

### High glucose increased ADAM17 and GRO-α/CXCR2 expression in HUVECs

**Fig. 1:**
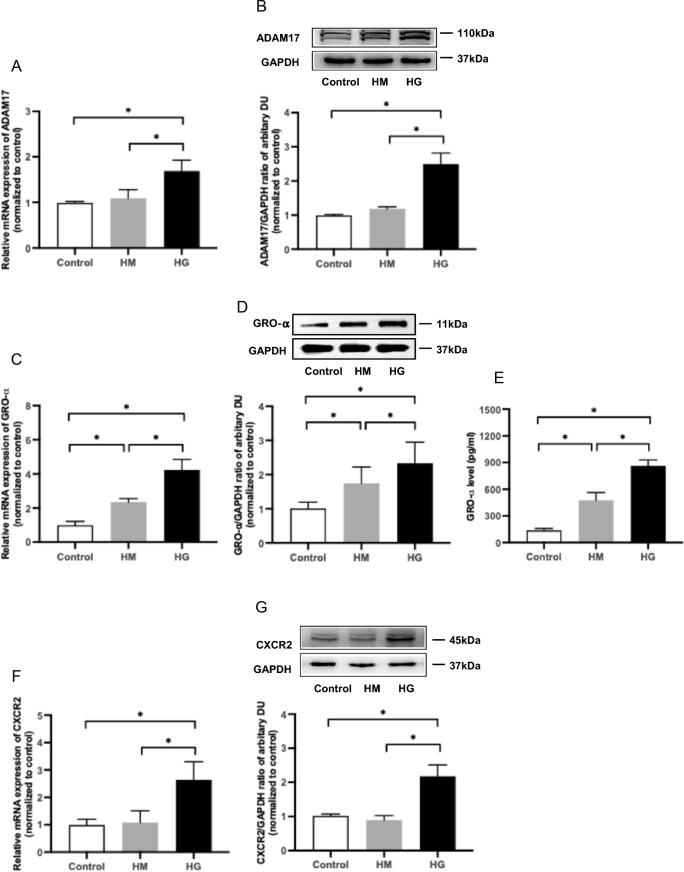
Effect of high glucose on ADAM17, GRO-α and CXCR2 expression in HUVECs. HUVECs were treated with SFM (control), 2.5% d-Mannitol (HM), or 2.5% d-Glucose (HG) for 24 h. ADAM17 (**A**), GRO-α **(C**) and CXCR2 (**F**) gene expression were measured by real-time PCR. Protein expression of ADAM17 (**B**), GRO-α (**D**) and CXCR2 (**G**) were assessed by western blotting and secreted GRO-α (**E**) in cell supernatants were measured by ELISA. Data were expressed as mean ± SD of three samples. ^*^P < 0.05

Incubation of HUVECs with 2.5% d-Glucose for 24 hours significantly increased ADAM17 gene and protein expression when compared to cells treated with SFM or 2.5% d-mannitol (*P* < 0.05 for all, Fig. 1A, B). GRO-α gene and protein expression, as well as secretion in HUVECs increased significantly when incubated with 2.5% d-mannitol and the elevation was further amplified in cells treated with 2.5% d-Glucose (*P* < 0.05 for all, Fig. 1C–E). High glucose increased CXCR2 gene and protein expression in HUVECs by 2.55 and 2.05 folds respectively when compared to SFM (*P* < 0.05 for all), whilst 2.5% mannitol did not affect CXCR2 expression significantly (Fig. 1F, G).

### ADAM17 gene-silencing abolished high glucose-mediated increase in GRO-α/CXCR2 expression in HUVECs

Gene-silencing with ADAM17-specific siRNA was associated with approximately 66.6%, 64.0% and 56.1% reduction in ADAM17 transcript in HUVECs under basal condition, or treated with a high concentration of mannitol or glucose, respectively (*P* < 0.05 for all, Figure S1). There were no significant changes in ADAM17 mRNA expression in cells incubated with scramble siRNA (*P* > 0.05 for all, Figure S1).

**Fig. 2:**
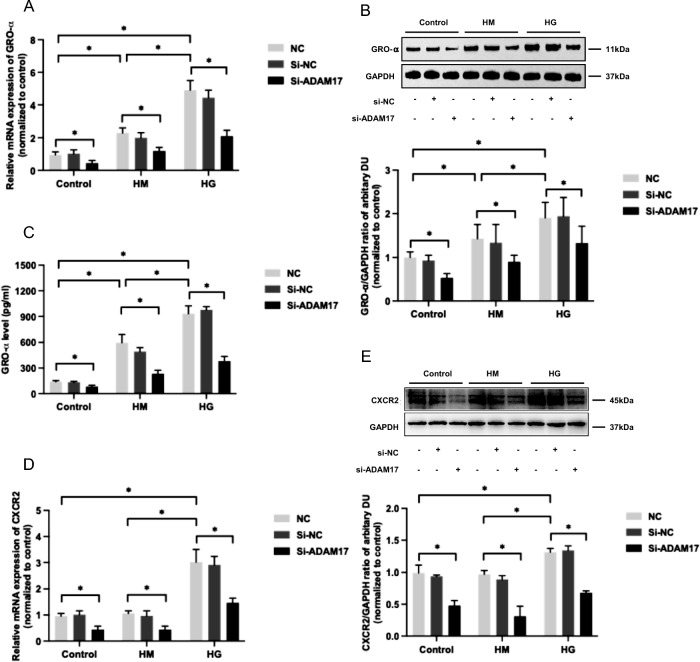
Effect of ADAM17 gene silencing on GRO-α and CXCR2 expression in HUVECs treated with high glucose. HUVECs were treated with ADAM17 siRNA (Si-ADAM17), scramble siRNA (Si-NC) or an equal volume of transfection reagent (NC) under incubation of SFM (control), 2.5% d-Mannitol (HM), or 2.5% d-Glucose (HG) for 24 h. GRO-α gene (**A**) and protein (**B**) expression, as well as secretion (**C**) were measured by real-time PCR, western blotting and ELISA respectively. CXCR2 gene expression (**D**) and protein expression (**E**) were assessed by real-time PCR and western blotting respectively. Data were expressed as mean ± SD of 3 samples. ^*^P < 0.05

ADAM17-specific siRNA was associated with a significant decrease in GRO-α mRNA and protein expression, as well as its secretion in cells cultured with SFM (*P* < 0.05 for all, Fig. 2A–C). It also attenuated the increase of GRO-α mRNA and protein expression in cells treated with either a high concentration of mannitol or glucose (*P* < 0.05 for all, Fig. 2A–C).

Suppression of ADAM17 was accompanied by 63.4% and 54.8% reduction in CXCR2 mRNA and protein expression respectively in cells under basal condition, and similar changes were observed in cells treated by 2.5% d-glucose or 2.5% d-mannitol (*P* < 0.05 for all, Fig. 2D, E). ADAM17 gene silencing abolished high glucose-mediated increase in CXCR2 mRNA and protein expression (*P* < 0.05 for all, Fig. 2D, E).

### ADAM17 gene-silencing suppressed high glucose-induced elevation of VEGF secretion by HUVECs

**Fig. 3:**
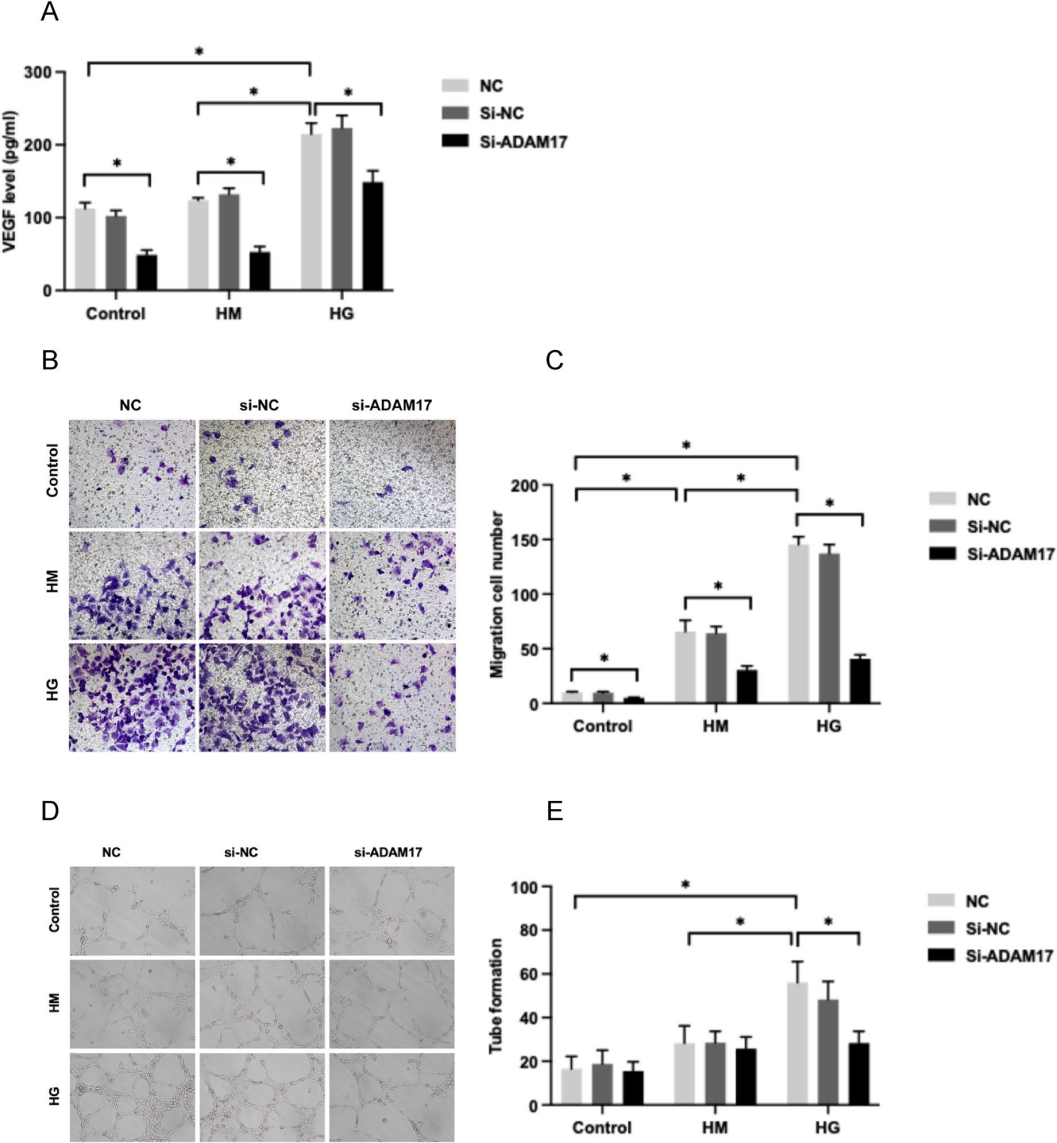
Effect of ADAM17 gene silencing on VEGF expression, migration and tube formation in HUVECs treated with high glucose. HUVECs were treated with ADAM17 siRNA (Si-ADAM17), scramble siRNA (Si-NC) or an equal volume of transfection reagent (NC) under incubation of SFM (control), 2.5% d-Mannitol (HM), or 2.5% d-Glucose (HG) for 24 h. **A** VEGF concentration in cellular supernatant was measured by ELISA. **B**, **C** Cell migration was assessed by migration assay. **D**, **E** Tube formation of HUVECs was determined by Matrigel. Data were expressed as mean ± SD of 3 samples. ^*^P < 0.05

2.5% d-glucose significantly increased the secretion of angiogenic marker VEGF by HUVECs compared to SFM or mannitol (*P* < 0.05, Fig. 3A). Incubation of ADAM17-specific siRNA almost abolished high glucose-induced increase in VEGF secretion by HUVECs (*P* < 0.05, Fig. 3A).

### ADAM17 gene-silencing attenuated high glucose-induced HUVECs migration

2.5% d-mannitol promoted migration of HUVECs and the effect was more pronounced in cells treated with 2.5% d-glucose (*P* < 0.05 for all, Fig. 3B, C). Incubation of ADAM17-specific siRNA partially decreased both hyperosmotic condition and high glucose-mediated HUVECs migration (*P* < 0.05 for all, Fig. 3B, C).

### Suppression of ADAM17 attenuated high glucose-induced tube formation in HUVECs

High glucose enhanced tube formation in HUVECs as assessed by Matrigel assay. Inhibition of ADAM17 by using specific siRNA ameliorated the angiogenic response of endothelial cells triggered by high glucose (*P* < 0.05 for all, Fig. 3D and E).

## Suppression of ADAM17 decreased high glucose-mediated increase of IL-6 and TNF-α secretion by HUVECs

**Fig. 4:**
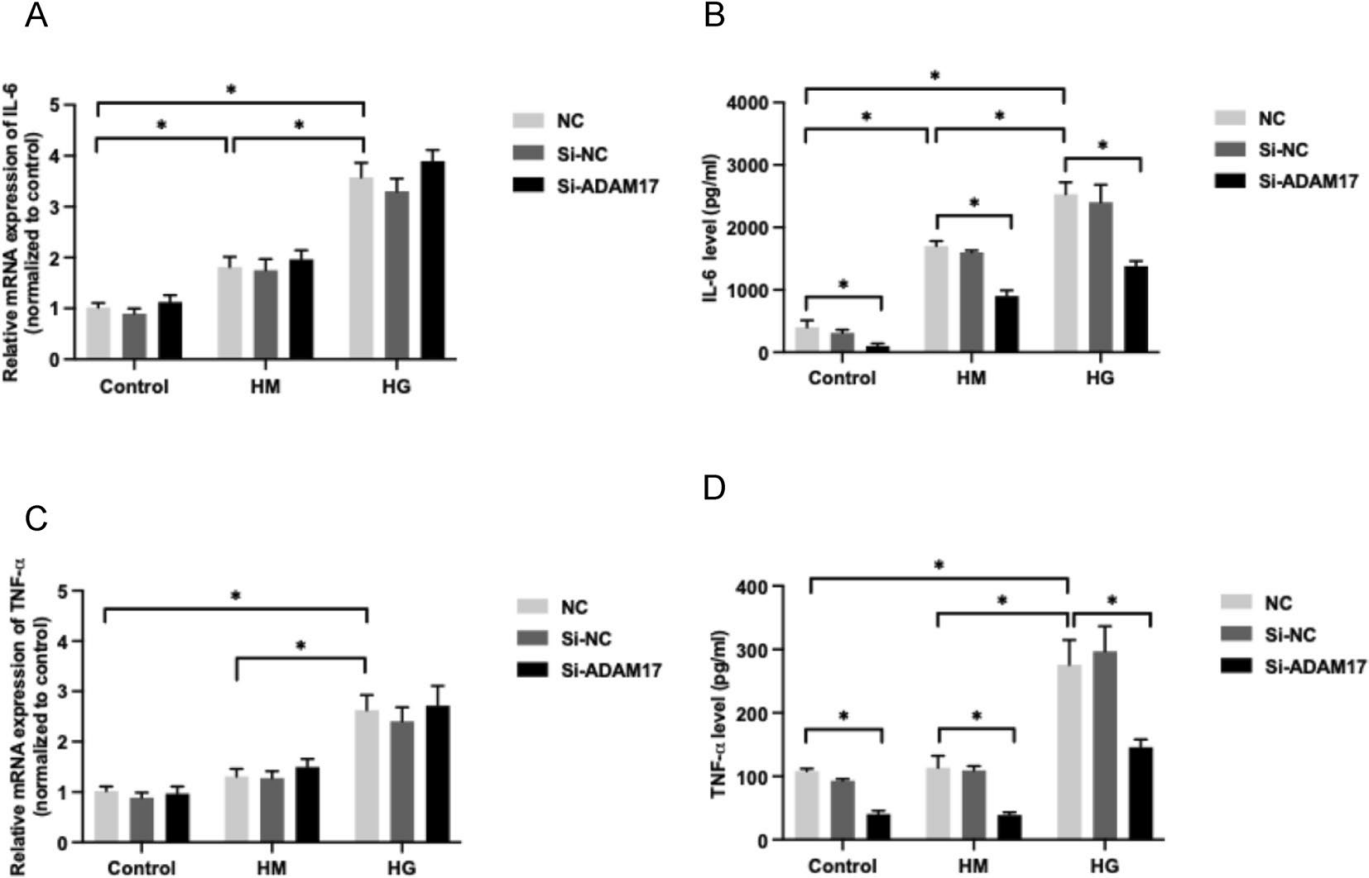
Effect of ADAM17 gene silencing on IL-6 and TNF-α expression in HUVECs treated with high glucose. HUVECs were treated with ADAM17 siRNA (Si-ADAM17), scramble siRNA (Si-NC) or an equal volume of transfection reagent (NC) under incubation of SFM (control), 2.5% d-Mannitol (HM), or 2.5% d-Glucose (HG) for 24 h. The mRNA expression of IL-6 (**A**) and TNF-α (**C**) were measured by real-time PCR. IL-6 (**B**) and TNF-α (**D**) concentrations in the cellular supernatant were measured by ELISA. Data were expressed as mean ± SD of 3 samples. ^*^P < 0.05

2.5% d-glucose significantly increased the mRNA expression and secretion of inflammatory cytokines of IL-6 and TNF-α in HUVECs (*P* < 0.05 for all, Fig. 4A–D). Incubation of ADAM17-specific siRNA significantly reduced the increase of IL-6 and TNF-α secretion mediated by high glucose in HUVECs (*P* < 0.05 for all), but it did not alter the mRNA expression of both cytokines (*P* > 0.05 for all, Fig. 4A–D).

## Discussion

Angiogenesis is a common characteristic of peritoneal structural changes during long-term PD. It is demonstrated as increased vessel density and abnormal vascular permeability, which are associated with increased effective peritoneal filtering surface area and elevated transport rate of small molecules [15]. Peritoneal inflammation is shown as enhanced infiltration of inflammatory cells and thus elevated production of cytokines including IL-6, IL-8 and TNF-α in the peritoneum. Peritoneal angiogenesis and inflammation, which interact with each other, have been reported to be the major pathological mechanisms responsible for the declined peritoneal function of ultrafiltration during the maintenance PD [4, 16]. However, prevention and intervention of peritoneal angiogenesis and inflammation in PD patients remain to be an unmet clinical need.

In the current study, we demonstrated that ADAM17, a zinc-dependent transmembrane multidomain metalloproteinase, was up-regulated in cultured HUVECs treated with high glucose. The induction of ADAM17 was accompanied by enhanced tube formation and elevated secretion of inflammatory cytokines. Inhibition of ADAM17 attenuated high glucose induced angiogenic and inflammatory responses of HUVECs, partly through suppression of GRO-α/CXCR2 expression.

High concentration of glucose-containing in conventional PD solutions, which provides an osmotic pressure gradient for dialysis, is a recognized risk factor of peritoneal angiogenesis and inflammation. Consistent with previous reports [17, 18], herein we found that high glucose-stimulated the secretion of VEGF, IL-6 and TNF-α in HUVECs in vitro, which was accompanied by enhanced cell migration and enhanced tube formation. VEGF, also known as vascular permeability factor, plays an important role in peritoneal angiogenesis by promoting vascular endothelial proliferation, stimulating vascularization, increasing vascular permeability and maintaining the normal state and integrity of blood vessels [15]. In the setting of PD, peritoneal vascular endothelial cells are not only the main target cells of VEGF but they can also synthesize VEGF themselves [19]. IL-6 is a central player in modulating peritoneal inflammation. It is suggested that IL-6, working together with soluble IL-6 receptor, could stimulate the synthesis of a variate of proinflammatory cytokines and chemokines, and thus amplify the inflammatory responses in the peritoneal cavity [20]. Recent studies from our research group and others found that IL-6 trans-signaling linked inflammation with angiogenesis in the peritoneal membrane by promoting the production of VEGF [21–23]. TNF-α is also a recognized proinflammatory cytokine that contributes to peritoneal angiogenesis through activation of angiopoietin-2/Tie2 signaling and other molecules [14].

The mechanisms of how high glucose-induced the production of VEGF, IL-6 and TNF-α in endothelial cells were not fully understood. In the current study, for the first time we demonstrated that incubation of HUVECs with 2.5% d-glucose in vitro increased the expression of ADAM17, and gene-silencing of ADAM17 attenuated high glucose-induced VEGF secretion, as well as IL-6 and TNF-α protein production. ADAM17 is a membrane-bound protease that sheds the extracellular domain of various receptors or their ligands from the cell membrane and subsequently activates downstream signaling transduction pathways. The angiogenic function of ADAM17 has already been reported in various physiologic and pathological processes including embryogenesis, tumorigenesis, and tissue repair [24–26]. Moreover, ADAM17 is shown to be intensively involved in the development of inflammation as it is able to cleave and activate a serial of proinflammatory cytokines and their receptors including IL-6R and TNF-α [27, 28]. Indeed, we demonstrated that blockade of ADAM17 lowered the secretion of IL-6 and TNF-α protein in HUVECs, but it did not change the mRNA expression of both cytokines. The findings indicated that the activity of ADAM17 in angiogenesis and inflammation reactions of HUVECs in response to high glucose was via its function of latent cytokines cleavage.

We next demonstrated that high glucose increased the expression of GRO-α and its receptor CXCR2 in HUVECs. High glucose up-regulated CXCR2 expression independent of its hyperosmotic characteristic. Whilst the effect of high glucose on inducing GRO-α expression was partly mediated through its hyperosmotic property. Furthermore, we found that the blockade of ADAM17 abolished the increase of GRO-α/CXCR2 mediated by high glucose in HUVECs. The changes were accompanied by weakened tube formation and cell migration, as well as reduced production of VEGF, IL-6 and TNF-α in HUVECs. It suggested that the effect of ADMA17 in mediating high glucose-induced endothelial cell angiogenesis and inflammation was partially related to modulation of GRO-α/CXCR2 complex. Our data were in agreement with the findings of Lisi and co-investigators, and they demonstrated that GRO-α/CXCR2 system and ADAM17 correlated expression in sjögren’s syndrome [11]. GRO-α/CXCR2 axis has also been reported to be participated in the processes of angiogenesis and inflammation in the setting of tumorigenesis, thrombin-induced angiogenesis, as well as pancreatic infection, since blockade of CXCR2 could suppress angiogenesis and leukocyte recruitment, [7–9].

There are some limitations in the current study that need to be discussed. First of all, this is an in vitro study and the findings will need to be further confirmed in animal study. Moreover, we observed that inhibition of ADAM17 in HUVECs was associated with reduced expression of GRO-α/CXCR2. However, the function of GRO-α/CXCR2 was not confirmed by using specific inhibitors. So, we cannot exclude the possibility that the effect of ADAM17 on angiogenic and inflammatory responses of HUVECs may partly be attributed to other mechanisms.

In conclusion, in this in vitro study, we demonstrated that ADAM17 was involved in the pathogenesis of angiogenic and inflammatory responses in HUVECs in vitro. Inhibition of ADAM17 attenuated high glucose-induced angiogenesis and inflammation in endothelial cells, partly through down-regulation of GRO-α/CXCR2 expression. It provides novel experimental evidence for designing strategies to preserve peritoneal structural and functional integrities in the clinic.

## Supplementary Information

Below is the link to the electronic supplementary material.

Supplementary file1 (DOCX 15 kb)
