# Supplementary material for: Inhibition of ADAM17 attenuates high glucose-induced angiogenesis and inflammation in endothelial cells partly through down-regulation of GRO-α/CXCR2 expression: implications in peritoneal dialysis
Source: Clin Exp Nephrol. 2024 Sep 21;28(12):1232–40. doi: 10.1007/s10157-024-02546-y (PMC11621206; doi:10.1007/s10157-024-02546-y)
Supplement: Supplementary file 1 — Supplementary file1 (DOCX 15 kb) [file 10157_2024_2546_MOESM1_ESM.docx]

**Supplementary materials:**

1. **Supplementary figures**

**Figure S1. Effect of ADAM17 gene silencing on ADAM17 mRNA expression in HUVECs.**

HUVECs were treated with ADAM17 siRNA (Si-ADAM17), scramble siRNA (Si-NC) or equal volume of transfection reagent (NC) under incubation of SFM (control), 2.5% D-Mannitol (HM), or 2.5% D-Glucose (HG) for 24 hours. ADAM17 mRNA expression was assessed by real-time PCR.

Data were expressed as mean ± SD of 3 samples. ^*^ *P* < 0.05.

1. **Supplementary tables**

**Table S1. Sequences of the ADAM17 siRNA and scramble siRNA**

| *ADAM17 siRNA* | sense: 5'-GCUCUCAGACUACGAUAUUTT-3' |
| --- | --- |
|  | antisense: 5'-AAUAUCGUAGUCUGAGAGCTT-3' |
| *Scramble siRNA* | sense: 5'-UUCUCCGAACGUGUCACGUTT-3' |
|  | antisense: 5'-ACGUGACACGUUCGGAGAATT-3' |

**Table S2.** **Primer Sequences of the targeted genes**

| ADAM17 | Forward: 5'- AATCTCTGTCTCTGTTTCACCC - 3' |
| --- | --- |
|  | Reverse: 5'- AAAGGGTTTGATAATGCGAACC - 3' |
| GRO-α | Forward: 5'- AAGAACATCCAAAGTGTGAACG - 3' |
|  | Reverse: 5'- CACTGTTCAGCATCTTTTCGAT - 3' |
| CXCR2 | Forward: 5'- AAGGTGAATGGCTGGATTTTTG- 3' |
|  | Reverse: 5'- CCCAGATGCTGAGACATATGAA - 3' |
| IL-6 | Forward: 5'- CTTCGGTCCAGTTGCCTTCT -3'  Reverse: 5'- GCTCTGGCTTGTTCCTCACT -3' |
| TNF-α | Forward: 5'- GTGACAAGCCTGTAGCCCAT -3'  Reverse: 5'- CAGACTCGGCAAAGTCGAGA-3' |
| GAPDH | Forward: 5'- CAGGAGGCATTGCTGATGAT- 3' |
|  | Reverse: 5'- GAAGGCTGGGGCTCATTT- 3' |
